# Pain Modulation after Oromucosal Cannabinoid Spray (SATIVEX^®^) in Patients with Multiple Sclerosis: A Study with Quantitative Sensory Testing and Laser-Evoked Potentials

**DOI:** 10.3390/medicines5030059

**Published:** 2018-06-21

**Authors:** Mara Turri, Francesco Teatini, Francesco Donato, Giampietro Zanette, Valeria Tugnoli, Luciano Deotto, Bruno Bonetti, Giovanna Squintani

**Affiliations:** 1 Department of Neurology, Central Hospital of Bolzano, 39100 Bolzano, Italy; francesco.teatini@sabes.it; 2 Department of Neurology, SS Giovanni e Paolo Hospital, 30122 Venice, Italy; donato.fnc@gmail.com; 3 Department of Neurology, Casa di Cura Pederzoli, 37019 Peschiera del Garda, VR, Italy; gi.zanette@libero.it; 4 Neurology Unit, Department of Neuroscience and Rehabilitation, S. Anna Hospital, 44124 Ferrara, Italy; v.tugnoli@ospfe.it; 5 Neurology Unit, Department of Neuroscience, AOUI Verona, 37126 Verona, Italy; luciano.deotto@gmail.it (L.D.); bruno.bonetti@univr.it (B.B.); giovannamaddalena.squintani@aovr.veneto.it (G.S.)

**Keywords:** pain, multiple sclerosis, oromucosal cannabinoid spray, quantitative sensory testing, laser-evoked potentials

## Abstract

**Background.** Delta-9-tetrahydrocannabinol (THC)/cannabidiol (CBD) (nabiximols or Sativex^®^) is an oromucosal spray formulation containing THC and CBD at an approximately 1:1 fixed ratio. Its administration for the treatment of pain in patients with multiple sclerosis (MS) has been established. MS patients generally complain of different kinds of pain, including spasticity-related and neuropathic pain. In this study, we compared and evaluated pain modulation and thermal/pain threshold of MS patients before and after THC/CBD administration. **Methods.** 19 MS patients underwent clinical examination, numerical rating scale (NRS), quantitative sensory testing (QST), and laser-evoked potentials (LEPs) before and after 1 month of therapy. Psychophysiological and neurophysiological data were compared to sex- and age-matched controls. **Results.** Patients reported a significant reduction in pain. We found statistically significant differences in LEP parameters between patients and controls but no significant change in LEP measures after THC/CBD therapy. Cold and heat detection thresholds were altered in patients but did not change after THC/CBD therapy. There was a significant increase in cold pain threshold by hand stimulation and a significant reduction in abnormal cold perception thresholds. **Conclusions.** Our results indicate that Sativex^®^ therapy provides pain relief in MS patients and suggest that it might modulate peripheral cold-sensitive TRP channels.

## 1. Introduction

Pain is a key symptom in patients affected by multiple sclerosis (MS) [1,2] and poses a considerable burden on quality of life [3] and disability [4]. Its prevalence varies between 29 and 86% [5] depending on the criteria defining the different types of MS-related pain and according to location, duration (paroxysmal, chronic) or presumed pain pathophysiology (central neuropathic or musculoskeletal) [6,7]. Spasticity-related pain, together with painful tonic spasms, is believed to be of mixed neuropathic and nociceptive origin [7] and to result from either corticospinal system disinhibition or chronic activation of nociceptive afferents [8]. 

Because of the limited response to current therapeutic options, exogenous cannabinoids have attracted increasing interest for the treatment of peripheral [9], central [10], neuropathic, and cancer pain [11]. Nabiximols (Sativex^®^) have been demonstrated as effective in MS-related pain syndromes [12,13,14] and approved in Italy since 2013 for symptomatic treatment of moderate-to-severe MS-related spasticity symptoms in adult patients refractory to other antispastic drugs [15]. Sativex^®^ is an oromucosal spray formulation derived from the *Cannabis sativa* plant, which contains delta-9-tetrahydrocannabinol (THC) and cannabidiol (CBD) in a nearly 1:1 ratio. THC is the main active substance, acting as a partial agonist at CB1 and CB2 receptors (CB1R and CB2R). Unlike traditional neurotransmitters, endogenous cannabinoids act as retrograde synaptic messengers, as they are released postsynaptically and activate CB receptors presynaptically. They exert their action by suppressing neurotransmitter release and modulating the excitatory effects of glutamate and inhibitory function of inter-neuronal gamma-aminobutyric acid [16].

The majority of these effects are mediated by CB1R, located in several pain areas such as the periaqueductal gray matter, spinal dorsal horn, and dorsal root ganglia neurons [17]. They contribute predominantly to antihyperalgesic effects in animal models [18]. CB2R, more diffusely distributed in peripheral tissues (immune organs and mast cells in particular) [17], has shown major efficacy in modulating pain of inflammatory origin [19,20]. Cannabinoids may also interact with many other neurotransmitters, including dopamine, acetylcholine, serotonin, and opioids [21]. Randomized controlled trials and observational studies testing the symptomatic efficacy of oral cannabinoids for relieving pain and spasticity in MS patients have generally evaluated response to treatment through subjective outcome measures: self-administered quality-of-life questionnaires and visual or numerical scales such as the Modified Ashworth Scale (MAS) for spasticity, or a numerical rating scale (NRS) for pain or spasticity [12,22,23,24].

The most reliable neurophysiological method to detect damage to nociceptive fibers in patients with neuropathic pain is laser-evoked potentials (LEPs) [25]. Also quantitative sensory testing (QST), a psychophysical exam, can assess small fiber function and may be appropriate to quantify positive sensory phenomena like mechanical and thermal allodynia and hyperalgesia, which may help characterize painful neuropathic syndromes and predict or monitor treatment effects [25,26].

Few studies to date have evaluated neurophysiological and psychophysiological data to assess pain modulation after cannabinoid intake [27]. The aim of the present study was to examine via psychophysiological and neurophysiological testing the effects of oromucosal THC/CBD spray (Sativex^®^) on the modulation of pain and thermal/pain thresholds in MS patients. 

## 2. Materials and Methods 

### 2.1. Patients 

A total of 28 MS outpatients were recruited. Inclusion criteria were diagnosis of MS according to Polman criteria [28], presence of chronic pain, spasticity recalcitrant to other drugs, age > 18 and <65 years. Exclusion criteria were any modification of ongoing therapy within the past 3 months, relapses in the 6 months prior to and during the study, high-dosage steroids in the last 6 months, pregnancy, severe kidney/liver disease, and history of drug abuse or mental disorder. Table 1 presents the characteristics of the study sample. 

All patients underwent thorough clinical examination, QST, and LEPs before and after 1 month of treatment with oromucosal THC/CBD. Pain was quantified using an 11-point numerical rating scale (NRS) (0 denotes no pain, 10 denotes worst possible pain). Psychophysical and neurophysiological test data were compared to 20 healthy controls sex- and age-matched to patient population. A definite diagnosis of neuropathic pain was established based on patient history and clinical findings, Douleur Neuropathique 4 (DN4) Questionnaire, and laboratory tests (including magnetic resonance imaging and neurophysiology) [8,29]. Patients with musculoskeletal or low back pain were considered to have nociceptive pain; alternately, coexistence of neuropathic and nociceptive pain was categorized as mixed [7]. The study was approved by the Local Ethics Committee of Borgo Trento Hospital; all patients gave their written, informed consent (Approval code: 17894). 

### 2.2. Neurophysiological and Psychophysiological Testing

#### 2.2.1. Laser-Evoked Potentials (LEPs)

Patients wore protective goggles and were seated in a comfortable chair in a warm, semi-darkened room. They were instructed to keep their eyes open while fixing upright and were asked to mentally count the number of laser stimulations to remain alert. The dorsa of the dominant hand and of both feet were stimulated by delivery of Nd:YAP laser (Electronic Engineering, Florence, Italy) (stimulation parameters: beam diameter 5 mm, pulse duration 5 ms, wave length 1.4 μm, stimulus intensity at pain threshold equivalent to a maximum energy of 6 joule) at pain threshold. Laser impact was visualized with a He–Ne laser beam, and the site of stimulation was slightly changed at each stimulus to avoid nociceptor sensitization. 

The pain threshold was calculated using the limits method: a stimulus was delivered by increasing its energy until reaching the pain threshold. Painful stimuli were rated on an 11-point NRS: the pain threshold was defined as a laser stimulus intensity corresponding to point 4 on the NRS. This stimulus intensity was then maintained constant during the test. A total of 25 to 30 laser stimuli were delivered in each trial at random intervals of 20 to 30 s. Data analysis was made off-line. LEPs were recorded (Keypoint EMG equipment, Dantec, Skovlunde, Denmark) with surface disc Ag-AgCl electrodes positioned at Cz, Fz, T3/T4, with reference to the nose, according to the 10–20 International System (recording parameters: amplification 30–50 µVolt/division, low filter 0.2 Hz, high filter 100 Hz); ocular movement was monitored by electro-oculography (EOG), and ocular artifacts were excluded from analysis. Peak latency of the N2 and P2 waves and peak-to-peak amplitude of the N2/P2 complex were evaluated. 

#### 2.2.2. Quantitative Sensory Testing (QST)

QST was performed using a TSA–II Thermotest Apparatus (Medoc Ltd., Ramat Yishai, Israel). A Peltrier thermode, (32 × 32 mm^2^) was fastened on the dorsa of hands and feet with a Velcro strap. Thermal and thermal pain thresholds were determined using the method of limits. Starting from a baseline temperature of 32 °C, the thermode temperature was increased to determine warm and heatpain thresholds and decreased to determine cold and cold-pain-detection thresholds. Patients were asked to press a button when they perceived thermal sensation.

Thermal detection (DT) and pain thresholds (PT) were determined in the following order: cold detection threshold (CDT), heat detection threshold (HDT), cold pain threshold (CPT), and heat-pain threshold (HPT). All thresholds were obtained with ramped stimuli (1 °C/s for thermal thresholds, 1.5 °C/s for pain thresholds), which were stopped when the patient pressed a button. The inter-trial interval was 4 s for DT, 10 s for PT. The ramp back to baseline was 1 °C/s for DT, 4 °C/s for CPT, and 8 °C/s for HPT. Probe cut-off temperature was 0 and 50 °C. Four consecutive measures were taken at each site to determine DT, and three to determine PT. Data were averaged to define cold, warm, heat, and cold-induced pain thresholds. 

### 2.3. Statistical Analysis

The NRS score for pain before (T0) and after therapy (T1) was compared using the non-parametric Wilcoxon test. Within each group of subjects, [controls (C), patients before therapy (PT0) and after therapy (PT1)], LEP N2-P2 complex amplitude, and N2 latency were compared using one-way analysis of variance (ANOVA) with groups as between-subject factor. Bonferroni correction was then applied for post-hoc multiple comparisons. Patient data at T0 and T1 were compared with paired Student’s *t* test. QST DT (CDT and HDT) and PT (CPT and HPT) between the two groups were compared with the non-parametric Kruskal–Wallis test; the Wilcoxon test was used to compare patient data at T0 and T1. As they had normal distribution, LEP latency and amplitude are presented as mean and standard deviation (SD), while QST CDT, HDT, CPT, and HPT are expressed as the median and 5th–95th percentile. 

We also calculated the number of abnormal measurements of LEP and QST parameters in the patients. LEP latencies or amplitudes at T0 and T1 were classified as abnormal when they were either absent or their values were outside the normal limits for latencies (mean + 2SD) and/or amplitudes (mean − 2SD). CDT and HPT were considered abnormal when the values were under the 5th percentile of normal values; HDT and CPT were considered abnormal when they exceeded the 95th percentile. We compared the rate of abnormal measurements at T0 and T1 using the chi-square test to see whether there was a difference in frequency before and after therapy. 

Unlike QST, which can also assess small fibers related nociceptive pain [30], LEPs are useful for assessing Aδ fiber pathway function in patients with neuropathic pain [25]. For this reason, we repeated the statistical analysis by selecting only those patients with pure neuropathic or mixed (both neuropathic and nociceptive) pain according to the definition of neuropathic pain [29]. 

Statistical analysis was performed using SPSS (Statistical Package for Social Sciences, Chicago, IL, USA); *p *< 0.05 was considered significant.

## 3. Results

In this group of 28 MS patients, 9 dropped out of the study because of drug abuse (*n* = 1, smoked marijuana abuse), relapse during 1-month Sativex^®^ therapy (*n* = 1), lack of compliance with neurophysiological studies (*n* = 4), and intolerable adverse events (dizziness) (*n* = 3, doses: 4 puffs for one and 6 puffs for two patients); among 19 MS patients who entered in the final analysis, 8 presented neuropathic pain, 6 nociceptive pain, and 5 mixed pain. 

All patients gradually increased their dose of oromucosal spray of Sativex^®^ until they achieved a satisfactory number of administrations per day (mean puffs/day 6.9 ± 1.9, range 4–11). Six patients reported mild side effects (dizziness in 4, drowsiness in 2, and lack of concentration in 2). A significant reduction in NRS score after drug therapy (from 6.61 to 3.55, *p *< 0.0001) was observed. If a 20% reduction in pain is considered clinically relevant, 14 patients (74%) responded to Sativex^®^ therapy. 

**Figure 1:**
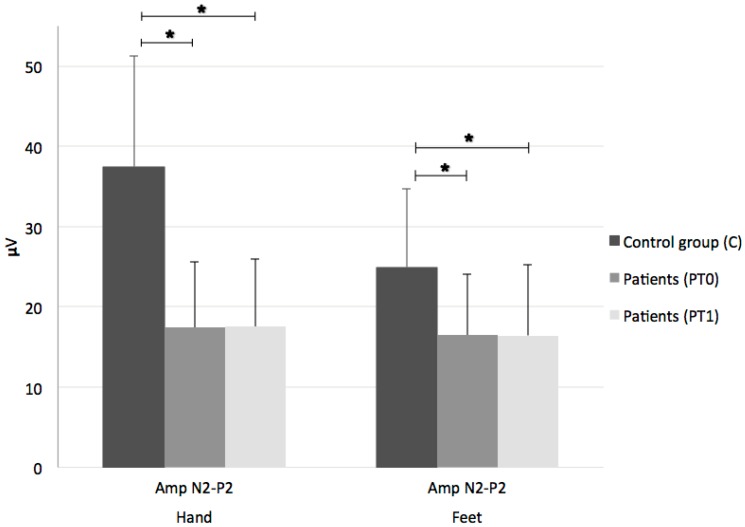
Laser-evoked potentials’ mean N2-P2 complex amplitudes for dominant hand and feet in control group (C) and patients before (PT0) and after (PT1) Sativex^®^. N2–P2 complex amplitudes in MS patients are significantly reduced in both dominant hand and feet compared to controls. No changes are observed after therapy. Amp: amplitude; *: statistically significant.

As compared to the controls, the MS patients had a significant reduction in LEP amplitude in either the dominant hand (mean N2–P2 complex amplitude (SD) 37.48 uV (13.79) for C vs. 17.41 uV (8.22) for PT0, F = 26,814, *p* < 0.0001) or the feet (mean N2-P2 complex amplitude (SD) 24.95 uV (9.75) for C vs. 16.46 uV (7.63) for PT0; F = 6,502, *p *= 0.006) (Figure 1). 

**Figure 2:**
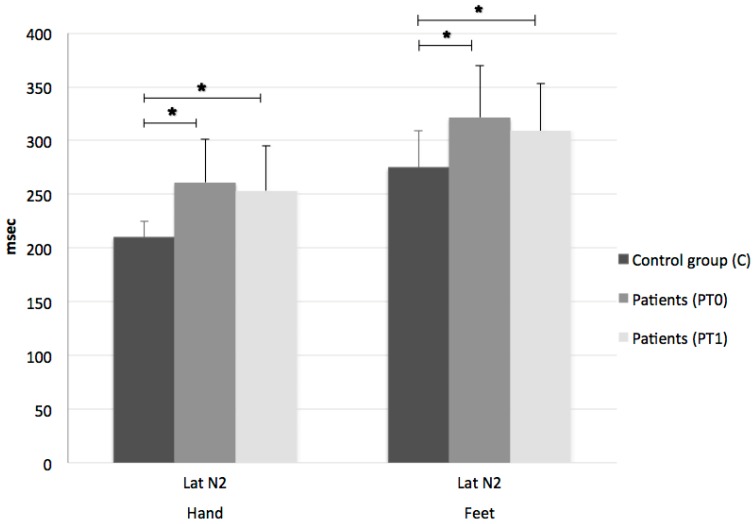
Laser-evoked potentials’ mean N2 latencies for dominant hand and feet in control group (C) and patients before (PT0) and after (PT1) Sativex^®^. N2 latencies in MS patients are significantly reduced in both dominant hand and feet compared to controls. No changes are observed after therapy. Lat: latency; *: statistically significant.

Significant differences were similarly found for N2 latency in either the dominant hand (mean N2 LEP latency (SD) 209.84 ms (14.70) for C vs. 261.18 ms (39.73) for PT0; F = 23,084, *p *< 0.0001) or the feet (mean N2 LEP latency (SD) 274.72 ms (34.12) for C vs. 321.67 ms (48.53) for PT0; F = 6,408, *p *= 0.002) (Figure 2). 

LEPs were inelicitable at the upper limbs in 11% of patients and at the lower limbs in 32%. Conversely, no significant change in LEP parameters was noted after drug administration (Figure 1 and Figure 2). 

Similar results were observed in the patients with neuropathic/mixed pain, with a significant decrease of amplitude and increased latency of the ‘neuropathic’ group compared to control group, in either the dominant hand or feet (dominant hand: mean N2–P2 complex amplitude (SD) 37.48 uV (13.79) for C vs. 18.08 uV (8.96) for PT0; F = 911,374, *p *< 0.0001; mean N2 latency (SD) 209.84 ms (14.7) for C vs. 245 ms (36.76) for PT0; F = 573,622, *p *< 0.0001; feet: mean N2–P2 complex amplitude (SD) 24.95 uV (9.75) for C vs. 12.85 uV (4.77) for PT0; F = 293,020, *p *< 0.0001; mean N2 latency (SD) 274.72 ms (34.12) for C vs. 332.5 ms (59.87); F = 752,471; *p *< 0.0001).

Also in patients with neuropathic or mixed pain, no change in LEP parameters was observed after Sativex^®^ therapy. 

**Figure 3:**
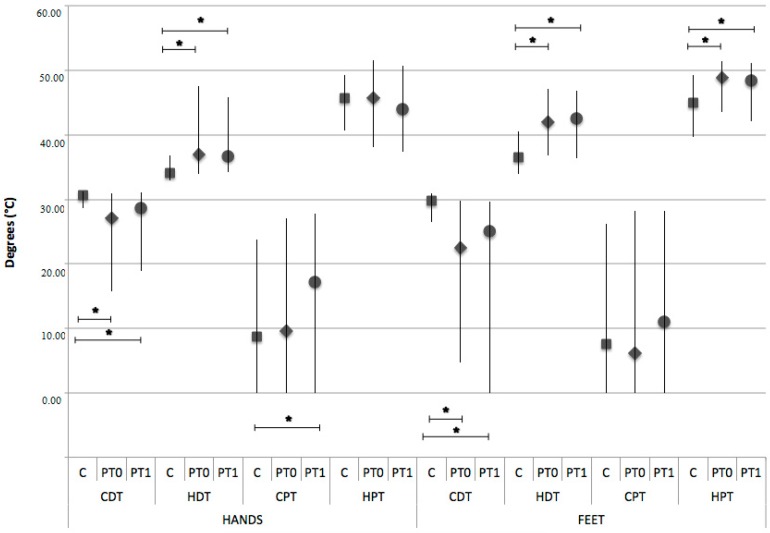
Quantitative Sensory Testing median values of cold and warm perception thresholds (CDT and HDT, respectively) and cold and heat pain thresholds (CPT and HPT, respectively) for hands and feet in control group (C) and patients before (PT0) and after (PT1) Sativex^®^. CDT and HDT for both hands and feet and HPT for feet are significantly altered in patients compared to controls and do not change after therapy. CPT measured by hand stimulation significantly rises after therapy. *: statistically significant.

QST cold and warm perception thresholds for both hands and feet and heat pain threshold for feet were also significantly altered in patients compared to controls [Hands: QST CDT median (5th–95th percentile) 30.67 °C (28.64–31.3) for C vs. 27.10 °C (15.77–30.9) for PT0; *p *< 0.0001; QST HDT median (5th–95th percentile) 34.07 °C (32.97–36.88) for C vs. 36.95 °C (33.96–47.54) for PT0; *p *< 0.0001; Feet: QST CDT median (5th–95th percentile) 29.77 °C (26.5–30.93) for C vs. 22.45 °C (4.66–29.83) for PT0; *p *< 0.0001; QST HDT median (5th–95th percentile) 36.48 °C (33.95–40.61) for C vs. 42 °C (36.84–47.12) for PT0; *p *< 0.0001; QST HPT median (5th–95th percentile) 44.94 °C (39.75–49.25) for C vs. 48.9 °C (43.6–51.5) for PT0; *p *< 0.0001] and did not change after THC/CBD therapy (Figure 3). 

We observed a significant posttreatment difference between controls and patients in cold pain threshold as measured by hand stimulation (QST CPT median (5th–95th percentile) 8.67 (0–23.71) for C vs. 17.25 (0–27.8) for PT1; *p *= 0016) (Figure 3). 

**Figure 4:**
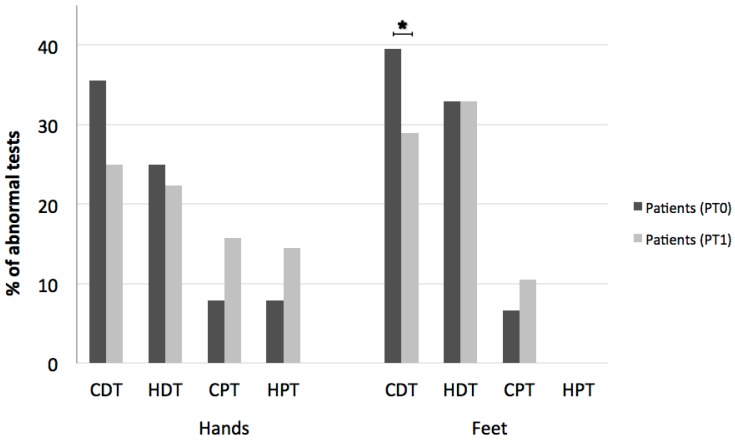
Percentages of abnormal Quantitative Sensory Testing for hands and feet in patients before (PT0) and after (PT1) THC/CBD therapy. A significant reduction in abnormal cold detection threshold (CDT) after Sativex^®^ is found in feet and a trend towards a reduction in abnormal CDT is also found in hands. *: statistically significant.

Comparison of the number of abnormal test results for patients before and after therapy showed a significant reduction in abnormal cold perception thresholds in feet (from 39.5 to 28.9%, *p *= 0.048) and a trend towards a reduction in abnormal CDT also in hands (from 35.5 to 25%, *p *= 0.06, not significant) (Figure 4). No differences in the other QST tests or LEP parameters were seen. No correlation between nabiximol dosage and expanded disability status scale (EDSS) change was recorded. 

## 4. Discussion 

Consistent with previous studies [31,32], our results show that Sativex^®^ therapy is effective for relieving pain, as seen in the significant reduction in the NRS scores. Furthermore, a significant reduction in amplitude and increased latency, probably caused by conduction block and demyelination damage of the spinothalamic pathway, was observed in the MS patients as compared to controls. This observation is shared by other studies [27,33]. Conversely, we observed no change in LEP parameters after drug therapy. Like other studies [27], this lack of change may be linked to several different factors, including severe impairment of nociceptive pathways, high disease burden, and coexistence of nociceptive and neuropathic pain in MS patients. 

Other studies have demonstrated a significant decrease in the N2–P2 complex amplitude after tramadol injection in healthy volunteers [34] and a decrease in amplitude and an increase in latency after oral carbamazepine in patients with trigeminal neuralgia [35]. We argue that severe damage to the nociceptive system related to MS may prevent significant changes in LEP; otherwise, cannabinoids may exert modulation on nociceptive pathways different from that of opioids or anticonvulsants, as shown by the reported finding of a decrease in N2 latency in a MS pain-free group after nabimixols therapy [27], suggesting recovery of conduction along pain pathways. 

Concerning QST, our results demonstrated a significant reduction in CDT and an increase in HDT in patients compared to controls. Intriguingly, there was a significant increase in the hand CPT of patients after Sativex^®^, and a significant percentage reduction in abnormal CDT after therapy, although the difference did not reach statistical significance between patients before and after treatment. Only one randomized, double-blind, placebo-controlled, crossover trial [36] compared QST thresholds before and after 3 weeks of dronabinol treatment in MS patients affected by central neuropathic pain, and obtained a significant reduction in pain without any changes in thermal and thermal pain thresholds. The discrepancy with our data may be due to different reasons, such as the effect of cannabidiol, the shorter treatment period in their trial, and the different selection criteria to enroll MS patients with central neuropathic pain.

Thermal sensation is known to be mediated by transient receptor potential (TRP) ion channels implicated in many physiological processes, including temperature sensation, pain, regulation of neurotransmitter release, and immune function. It is also established that THC and CBD may act as agonists of TRP channels, which is why they are also called ionotropic cannabinoid receptors to differentiate them from the classic CB1 and CB2 metabotropic receptors [37]. The sub-class TRPA1 are cold-sensitive cation channels that function as sensors of painful cold [38]. They are located predominantly in the nociceptive neurons of the peripheral nervous system (PNS) and may act as mediators of mechanical hyperalgesia and cold hypersensitivity [39,40]; their transcription is increased during inflammation and its suppression reduces hypersensitivity to cold temperature (cold allodynia) in rat models of inflammation and nerve injury [41]. Although the contribution of TRPA1 to thermosensation is controversial [38], TRPA1 has been implicated also in cold sensation in vitro and in vivo [42,43]. For these reasons, TRPA1 are important for the detection of noxious stimuli and pain transduction [44,45].

The mechanism of action of cannabinoids on TRP are multiple and complex, as some cannabinoids may indirectly suppress TRPV1 and TRPA effects on pain and inflammation by acting on CB1, but directly activate the TRP channel at higher concentrations [18,46]. Furthermore, non-psychotropic cannabinoids (such as CBD) can activate and desensitize TRPV1 and TRPA1 [47].

TRPM8, another member of the TRP superfamily, is also involved in thermosensation and has a role in the detection and transmission of cold stimuli [38,48] and cold hypersensitivity [49]. De Petrocellis and co-workers [50] demonstrated that certain phyocannabinoids (THC and CBD in particular) can efficaciously antagonize the effect of TRPM8 agonists. 

In our study, the increase in cold pain threshold in hands and the normalization of cold detection threshold in MS patients after therapy could reflect a cannabinoid-mediated modulation on either TRPA1 or TRPM8 channels, probably via activation (and consequent desensitization) of TRPA1 and inhibition of TRPM8, although the mechanisms of action of the single channels need to be elucidated [40]. If cooling increases the open probability of the TRPA1 channel [40], the fact that the patients felt noxious cold at higher temperatures could mean activation (and consequent desensitization) of this channel and, at the same time, an antagonistic effect on TRPM8 [48]. The lack of the same finding at the feet could be interpreted as a failure of Sativex^®^ to modulate the more severely damaged nociceptive pathways, as suggested by the high number of abnormal thermal detections and sensory impairment evidenced on clinical examination of the feet. Furthermore, although the cold pain threshold was increased (from 9.55 °C to 17.25 °C) in the patients after Sativex^®^ therapy, its value remained within the normal range (<23.71 °C) and they did not complain of thermal allodynia before or after drug intake. 

We acknowledge several limitations of our study: first, the small sample size and high EDSS; second, the lack of a placebo group, although Sativex^®^ efficacy for the relief of both nociceptive and neuropathic pain has been demonstrated in some controlled studies [10,51]; third, the short observation period, though the long-term efficacy of nabimixols has only recently been proved [43,52]. Further studies with larger patient samples and lower EDSS are needed to better clarify how Sativex^®^ relives pain symptoms in MS.

In summary, our study further corroborates evidence for the effectiveness of Sativex^®^ in reducing pain in MS patients. Furthermore, our findings suggest a possible direct effect of cannabinoids on TRP channels by modulating or desensitizing cold-channel functions, in particular TRPA1 and TRPM8. This underscores the importance of TRP channels as a therapeutic target by acting through peripheral desensitization and inhibition of sensory neurons [53]. Several TRP subtypes may not only function as an integral part of the endocannabinoid system but also represent promising molecular targets for pain alleviation, especially when upregulated/sensitized in pathological or inflammatory conditions [37].

## Figures and Tables

**Table 1:** Demographics and clinical features of multiple sclerosis (MS) patients.

Case Number	Age	Gender	MS Subtype	Disease Duration (years)	EDSS	MAS	NRS	Type of Pain
1	56	M	RR	9	4.5	0.3	6	*neuropathic* *
2	54	F	RR	33	5.5	0.1	6	*nociceptive*
3	46	M	PP	15	7.5	0.8	4	*nociceptive*
4	37	M	SP	18	6	1.8	7	*mixed* *
5	63	F	RR	4	5.5	1.3	4	*nociceptive*
6	48	F	SP	21	7.5	2.0	6	*neuropathic*
7	58	F	SP	13	5.5	1.3	5.5	*nociceptive* *
8	48	M	SP	7	5.5	0.8	6	*nociceptive* *
9	61	F	SP	30	6.5	1.1	2	*nociceptive*
10	48	F	RR	16	6	1.8	7	*neuropathic*
11	59	M	SP	23	5.5	1.3	4	*neuropathic* *
12	61	M	SP	10	6	1.3	3.5	*mixed* *
13	65	F	PP	43	6.5	1.3	10	*neuropathic*
14	63	F	SP	17	7	1.6	6	*nociceptive*
15	49	M	SP	3	5.5	0.8	10	*mixed*
16	56	F	SP	23	7.5	2.0	9	*mixed*
17	57	M	PP	26	8	2.3	4.5	*nociceptive*
18	57	M	RR	17	5.5	0.7	6	*neuropathic* *
19	57	F	SP	33	6	0.5	2	*neuropathic*
20	58	M	RR	17	6	0.8	9	*neuropathic*
21	47	F	RR	16	3.5	0.3	8	*neuropathic*
22	59	F	SP	36	4.5	0.8	8.5	*neuropathic* *
23	51	F	SP	20	7	1.2	3	*nociceptive* *
24	65	F	PP	8	6	1.3	8	*mixed*
25	59	F	SP	18	7.5	1.3	7	*mixed*
26	61	M	SP	19	7.5	1.3	10	*neuropathic*
27	44	F	RR	1	2	0.1	6	*mixed*
28	44	F	SP	5	5.5	0.2	7	*neuropathic*
Mean (SD)	54.7 (7.3)	11M/17F	8 RR/4 PP/16 SP	18 (10.5)	6 (1.31)	1.1 (0.6)	6.3 (2.3)	12 neuropathic 9 nociceptive 7 mixed

Legend: SD: standard deviation; F: female, M: male; RR: Relapsing Remitting; PP: Primary Progressive; SP: Secondary Progressive; EDSS: Expanded Disability Status Scale; MAS: Modified Ashworth Scale; NRS: Numerical Rating Scale. * Patients not included in the statistical analysis.
